# 

**Published:** 1888-02

**Authors:** 


					﻿Received following pamphlets:
How They were Converted. Homoeopathic League Tract,
No. 16.
The Homoeopathic Domestic Indicator.
Fine parts treating respectively on Materia Medica, Diphtheria, Croup,
Cholera, Toothache, and lastly, The External Application of Remedies.
These series of pamphlets were translated from the German by Dr. J. Foster,
and are published by Dr. Schwabe of the Leipsic Homoeopathic Pharmacy.
NOTICE.
All articles for publication, books for review, business communications, etc.,
must be sent to Editors, 1123 Spruce Street, Philadelphia.
Subscribers will please notice that this Journal will be sent until arrears
are paid and it is ordered discontinued.
Subscribers are respectfully requested to make all checks
< and money-orders payable to THE HOMOEOPATHIC
PHYSICIAN, and not to either of the Editors.
				

